# Supplementary material for: Risk factors for sacrococcygeal pilonidal sinus: a systematic review and meta-analysis supplemented by genetic causal assessment
Source: Front Surg. 2026 Jan 7;12:1718589. doi: 10.3389/fsurg.2025.1718589 (PMC12819706; doi:10.3389/fsurg.2025.1718589)
Supplement: Supplementary file 2 [file Datasheet2.zip › Supplementary Data 2/MR_pipeline_p5e-8/forest_plots/ebi-a-GCST90014023.finngen_R12_L12_PILONIDALCYST.pdf]

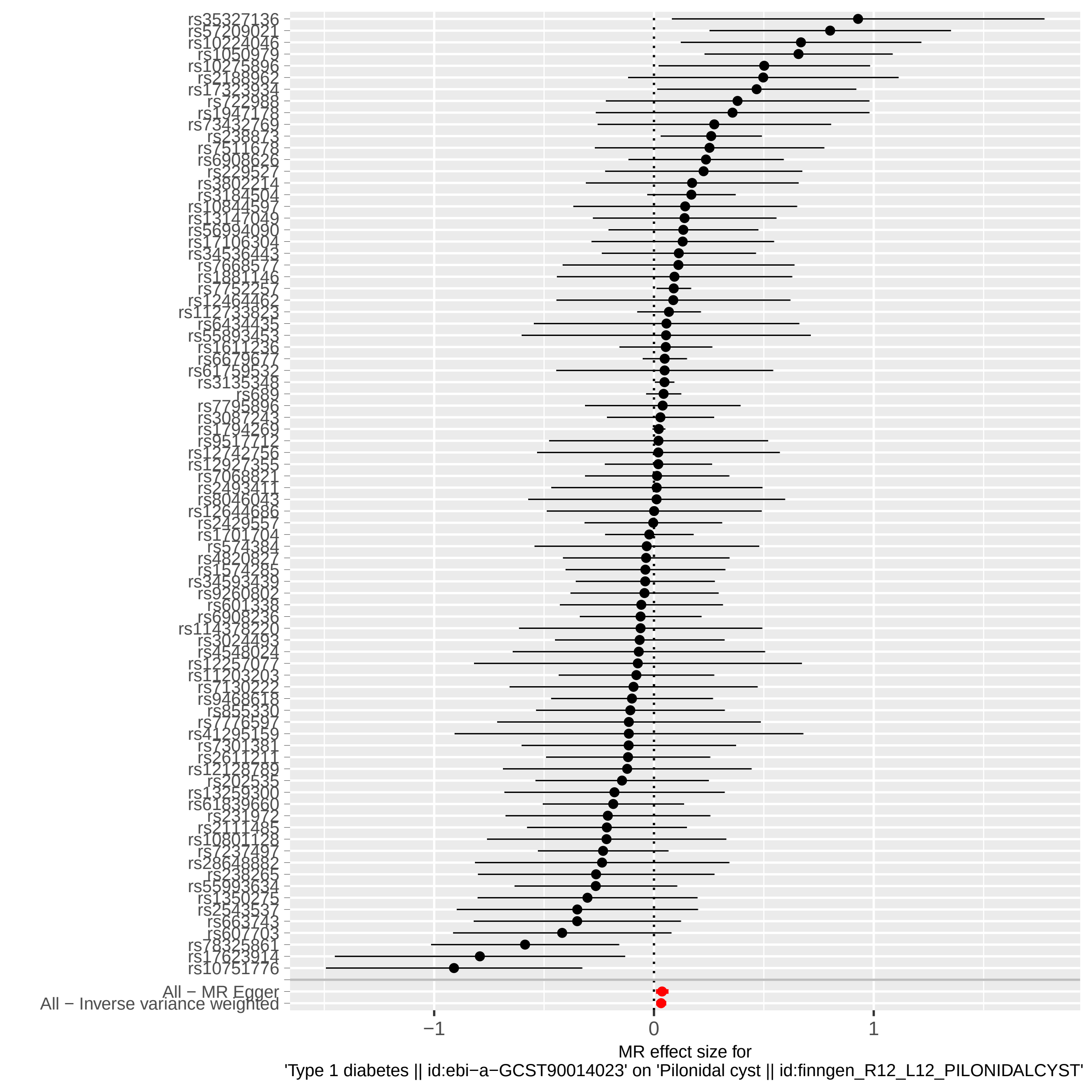

rs35327136
rs57209021
rs10224046
rs1050979
rs10275896
rs2188962
rs17323934
rs722988
rs1947178
rs73432769
rs238873
rs7511678
rs6908626
rs229527
rs3802214
rs3184504
rs10844597
rs13147049
rs56994090
rs17106304
rs34536443
rs7668577
rs1881146
rs7752257
rs12464462
rs112733823
rs6434435
rs55893453
rs1611236
rs6679677
rs61759532
rs3135348
rs689
rs7795896
rs3087243
rs1794269
rs9517712
rs12742756
rs12927355
rs7068821
rs2493411
rs8046043
rs12644686
rs2429557
rs1701704
rs574384
rs4820827
rs1574285
rs34593439
rs9260802
rs601338
rs6908236
rs114378220
rs3024493
rs4548024
rs12257077
rs11203203
rs7130222
rs9468618
rs855330
rs7776597
rs41295159
rs7301381
rs2611211
rs12128789
rs202535
rs13259300
rs61839660
rs231972
rs2111485
rs10801128
rs7237497
rs28648882
rs238265
rs55993634
rs1350275
rs2543537
rs663743
rs607703
rs78325861
rs17623914
rs10751776
All − MR Egger
All − Inverse variance weighted
−1
0
1
MR effect size for
'Type 1 diabetes || id:ebi−a−GCST90014023' on 'Pilonidal cyst || id:finngen_R12_L12_PILONIDALCYST'
